# Sheep nemabiome diversity and its response to anthelmintic treatment in Swedish sheep herds

**DOI:** 10.1186/s13071-021-04602-y

**Published:** 2021-02-18

**Authors:** Peter Halvarsson, Johan Höglund

**Affiliations:** Department of Biomedical Sciences and Veterinary Public Health, Swedish University of Agricultural Sciences, Section for Parasitology, Uppsala, Sweden

**Keywords:** PacBio, Gastrointestinal nematode, Livestock, Anthelmintics, Biodiversity, Diversity index

## Abstract

**Background:**

A novel way to study the species composition and diversity of nematode parasites in livestock is to perform deep sequencing on composite samples containing a mixture of different species. Herein we describe for the first time the nematode community structures (nemabiomes) inhabiting Swedish sheep and how these are/were affected by host age and recent anthelmintic treatments.

**Methods:**

A total of 158 fecal samples were collected (*n* = 35 in 2007 and *n* = 123 in 2013–2016) and cultured from groups of sheep on 61 commercial farms in the south-central part of the country where most animals are grazed. Among the samples, 2 × 44 (56%) were paired collections from the same groups pre- and post-treatment with anthelmintics such as macrocyclic lactones, benzimidazoles or levamisole. Samples were analyzed for their nemabiome using the PacBio platform followed by bioinformatic sequence analysis with SCATA. Species richness and diversity were calculated and analyzed in R.

**Results:**

Nematode ITS2 sequences were found in all larval culture samples except two, even though the fecal egg counts were below the McMaster threshold in 20 samples. Sequencing yielded, on average, 1008 sequences per sample. In total, 16 operational taxonomical units (OTU), all with ≥ 98 % identity to sequences in the NCBI database, were recognized. The OTUs found represented nematode species of which ten are commonly associated with sheep. Multiple species were identified in all pre-anthelmintic treatment larval culture samples. No effects on nematode diversity were found in relation to host age. On the other hand, recent anthelmintic treatment lowered species richness, especially after use of ivermectin and albendazole. Interestingly, despite zero egg counts after use of levamisole, these samples still contained nematode DNA and especially *H. contortus*.

**Conclusions:**

Our findings provide evidence that nemabiome analysis combined with diversity index analysis provides an objective methodology in the study of the efficacy of anthelmintic treatment as both high and low abundant species were detected.
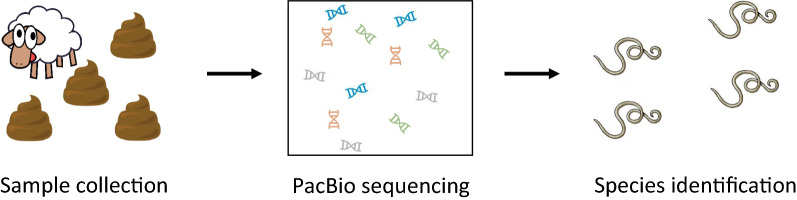

## Background

Infection with gastrointestinal nematode (GIN) parasites is well known globally as a major veterinary problem which contributes to a decline in the productivity of the global sheep industry [1]. Grazing sheep in Sweden are typically infected by a wide range of GIN with the majority belonging to the superfamily Trichostrongyloidea within the order Strongylida [2], among which some species such as *Haemonchus contortus* are considered more pathogenic than others [3]. Accordingly, parasite identification is fundamental for the improvement of sustainable parasite control strategies. The relative abundance of the different parasitic species present in sheep is driven by a wide range of factors. These include external factors such as climate and managerial factors affecting the exposure to the free living stages [4] and, not least, selection reinforced by use of anthelmintic compounds [5]. In addition, parasites are influenced by host immunity affected by previous exposure [6] and the presence of other animals that may act as reservoirs [7].

Traditional parasitological diagnostic techniques, based on microscopical examination of fecal eggs counts (FEC) and larval cultures, can provide rough measures of the nematode species or genus composition in sheep [8]. However, a disadvantage of these diagnostic tools is that they rely on trained experts, which are hard to find nowadays, but it is clear that they may have major constraints in terms of both sensitivity and specificity [9]. Thus, there is a need to utilize improved diagnostics for the investigation of complex nematode communities. Access to diagnostic instruments that could rapidly rank strongyle nematodes according to their relative contribution to mixed infections would represent a major advantage particularly in terms of the understanding of how selection by anthelmintics shapes nematode communities. This present need for new diagnostics is underscored by the increasing evidence of anthelmintic resistance (AR) and requests for evidence-based use of anthelmintic drugs in parasite control [10]. Although effective parasite control in general can be achieved through a combination of grazing management strategies and treatment with anthelmintics, this practice is under threat due to the increasing spread of resistance to these veterinary drugs [11].

For some time, the molecular identification of parasitic nematodes largely relied on amplification of the internal transcribed spacer two (ITS2) region located between the 5.8S and 28S subunits of the ribosome encoding genes [12]. Today several DNA-based tests are available for genotyping of GIN that offer the potential to detect, identify and quantify especially strongyle nematode parasites in ruminants [13–15]. However, these different technologies in general suffer from limitations in terms of the number genera and/or species that can be detected since they are usually tailored to the few parasites that are considered to be of particular interest and to which primer probe sets have been designed. The advent of deep amplicon sequencing using next-generation sequencing (NGS) platforms has generated new prospects and simplified the sequencing of gene amplicons in the study of microbial communities that usually exist as mixed infections within their hosts. Like for any microbe, NGS allows for the characterization of complex nematode communities and opens up new possibilities to identify community components even at low relative abundances at an unprecedented depth with minimal cost and labor. Recently the term “nemabiome” was created to describe the community structure of nematodes in ruminant livestock [16]. By using an Illumina-based deep amplicon next-generation pooled sequencing approach targeting the nematode ITS2 rRNA gene, a pipeline was developed which so far has been used to study the entire nemabiome in beef cattle [16], bison [17] and dairy cattle [18] in Canada and the US, as well as in UK sheep [19]. Similarly, the nemabiome of equines was recently studied using a slightly different methodology [20]. Combined, these studies have revealed detailed insights into the nematode diversity in each of these hosts using a truly non-invasive diagnostic approach.

In this article, we describe the species composition and diversity of GIN by studying the nemabiome in 158 sheep larval cultures collected on 61 commercial farms distributed across Sweden. The results were analyzed investigating how the nemabiomes were affected by (i) host age, (ii) in response to anthelmintic treatments and (iii) their long-term temporal effects. The study was conducted using data generated on the Pacific Biosciences (PacBio) platform, which for other microbial communities has been shown to produce less bias compared to other deep sequencing technologies [21]. The Operational Taxonomic Units (OTU) we refer to were distinguished using SCATA (https://scata.mykopat.slu.se/), which is a specifically designed analysis framework for the evaluation of sequenced tagged amplicons derived from eukaryotic communities.

## Methods

### Sample collection

**Fig. 1.**
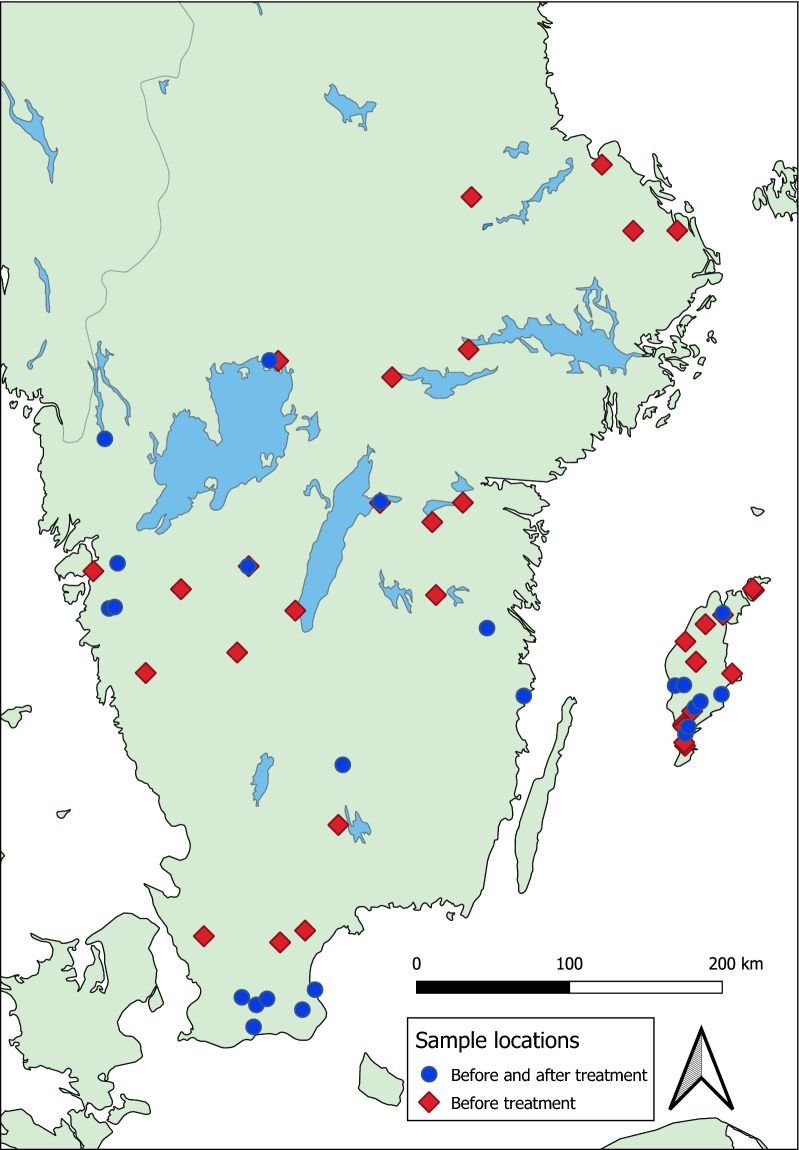
Map of sampling locations in Sweden. The map was created in QGIS 3.14 with Natural Earth vector data

The sampling was carried out either as part of random investigation or from commercial sheep farms suffering from recurrent problems with GIN. In total, 158 fecal samples were obtained from 19 farms during 2007 (*n* = 35 lambs) and on 42 farms between 2013 and 2016 (*n* = 123, of which 74 from adults and 49 from lambs) (Fig. 1). On each sampling occasion approximately two tablespoons of fresh feces was collected per rectum from each of 10 or 15 randomly selected animals (either ewes or lambs) in the flock. This was done by the farmer or a veterinarian at the Farm and Animal Health in Sweden. After treatment, the sheep were kept in their respective herd. Individual fecal samples were placed in marked zip-locked plastic bags after the air had been pressed out before sealing and then sent overnight by national post to the diagnostic laboratory (Vidilab AB).

On 44 of the sampling occasions, paired pre- and post-treatment samples were obtained, 24 paired fecal samples from adults and 20 from lambs, i.e. 88 samples. Among these, 26 groups were treated according to the recommended dose with the macrocylic lactone ivermectin (IVM), 13 with the benzimidazole albendazole (ABZ) and 5 with the imidazothiazole derivative levamisole (LEV). On ten farms the efficacy of several anthelmintics was tested in parallel using different groups of animals in the flock. The post-treatment collection was done from the same animals between 7 to 10 days after deworming in accordance with McKenna et al. [22]. These samples originated from routine monitoring programs and were collected by veterinarians; thus, they reflect the actual use of anthelmintics on sheep farms in Sweden.

### Parasitological investigation and DNA extraction

Upon arrival to the laboratory, feces were investigated for GIN strongyle eggs. The number of nematode eggs was first counted using a modified McMaster technology based on 3 g of feces and with a minimum diagnostic sensitivity of 50 nematode eggs per gram feces (EPG) as described previously [23].

Thereafter, subsamples of approximately 2 g feces were collected from each animal in all groups and pooled in separate plastic containers. After blending the pooled fresh feces with Vermiculite, the eggs were cultured for approximately 10 days in a moistened chamber at ≈20 °C [24]. Infective third-stage larvae (L3) were harvested using the Petri dish method, concentrated in a Falcon tube and collected into an Eppendorf tube before storage in a freezer at approximately − 18 °C [14]. DNA was then extracted from the larval cultures (one per group and sampling occasion) using the Nucleospin® DNA tissue kit (Macherey-Nagel, Düren, Germany) following the manufacturer's guidelines.

### Library preparations and sequencing

Nematode parasite ITS2 rDNA fragments were amplified using the NC1–NC2 primer pair [25] tagged with an 8-bp-long barcode [26] on the 5’ side of each primer. PCR was conducted in 50 µl reactions consisting of 0.2 µM dNTPs, 0.75 µM MgCl2, 1 µM each of NC1–NC2 primer pair, 2.5 µl DNA template and 1.25 U DreamTaq DNA polymerase (ThermoFisher Scientific, Waltham, MA, USA). The PCR cycling consisted of an initial denaturation for 3 min at 95 °C followed by 20–34 cycles of 30 s at 94 °C, 30 s at 55 °C and 30 s at 72 °C and finally 7 min at 72 °C where the number of PCR cycles for each sample was determined depending on template concentration. Each sample was purified using Agencourt AMPure beads (Beckman Coulter, Indianapolis, IN, USA), and the purified PCR product was measured fluorometrically (Qubit Fluorometer). The same amount of PCR product from each sample was pooled into four libraries. Sequence ligation to each library and sequencing on PacBio SMRT cell V3 RSII were performed by SciLifeLab, Uppsala, Sweden.

### Bioinformatic analysis

The raw sequence reads were processed using the SCATA bioinformatics pipeline (http://scata.mykopat.slu.se/). Sequences with an average quality score < 20 or with a score < 10 at any single position were removed. The primer and tag sequences were then removed from the remaining reads. Homopolymers were collapsed to 3 bp when OTUs could not be delimited based on differences in homopolymer regions [27], as current sequencing platforms show systematic sequencing errors in these regions [28]. During the clustering procedure, sequences were compared pairwise for similarity using USEARCH [29] with a minimum length set to 85% of the longest sequence. Pairwise alignments were scored using a mismatch penalty of 1, gap opening penalty of 0 and gap extension penalty of 1. Sequences were clustered into operational taxonomic units (OTUs) by single linkage clustering with a 98.5% sequence similarity required to be assigned to OTUs [30]. Read counts of all OTUs were converted to relative proportion of the total counts of each sample. After the SCATA pipeline, samples with < 200 reads were omitted and OTUs containing < 1% of the total reads per sample were filtered to reduce the background noise and background contamination. Each OTU was manually searched for similarity using NCBI BLAST. OTUs not representing nematodes were omitted from further analyses. For transparency, a scatterplot with number of reads per sample (Additional file 1: Fig. S1), unfiltered data and analyses can be found in the supplementary data file (Additional file 1: Tables S1, S2 and Fig. S2–S4).

### Statistical analysis

**Fig. 2:**
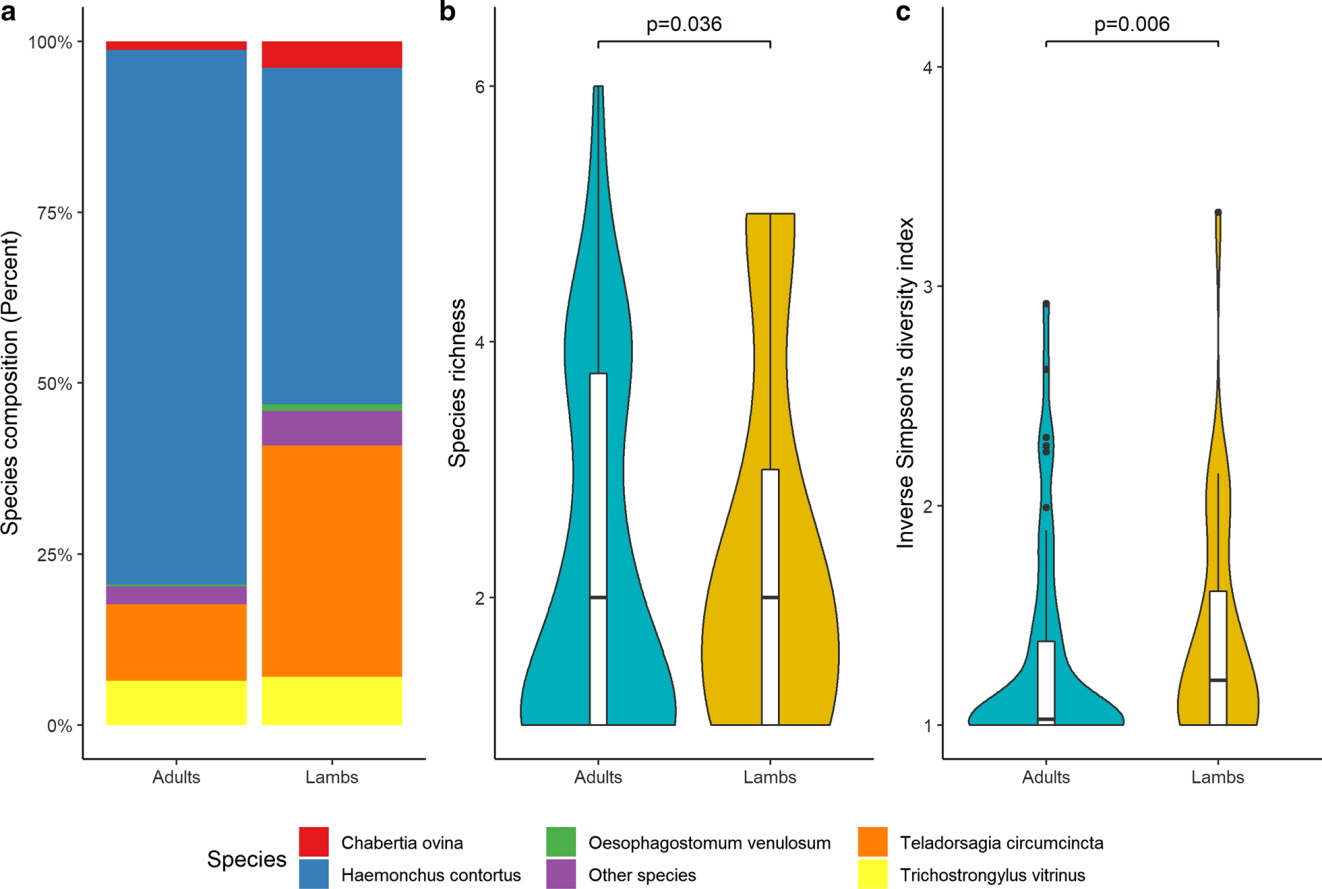
**a** Nemabiome composition for adults (*n* = 74) and lambs (*n* = 84). A difference in nematode species richness (**b**), and adults were dominated by a few species compared to lambs (**c**). Boxplots inside the violin plots display the median

**Fig. 3.**
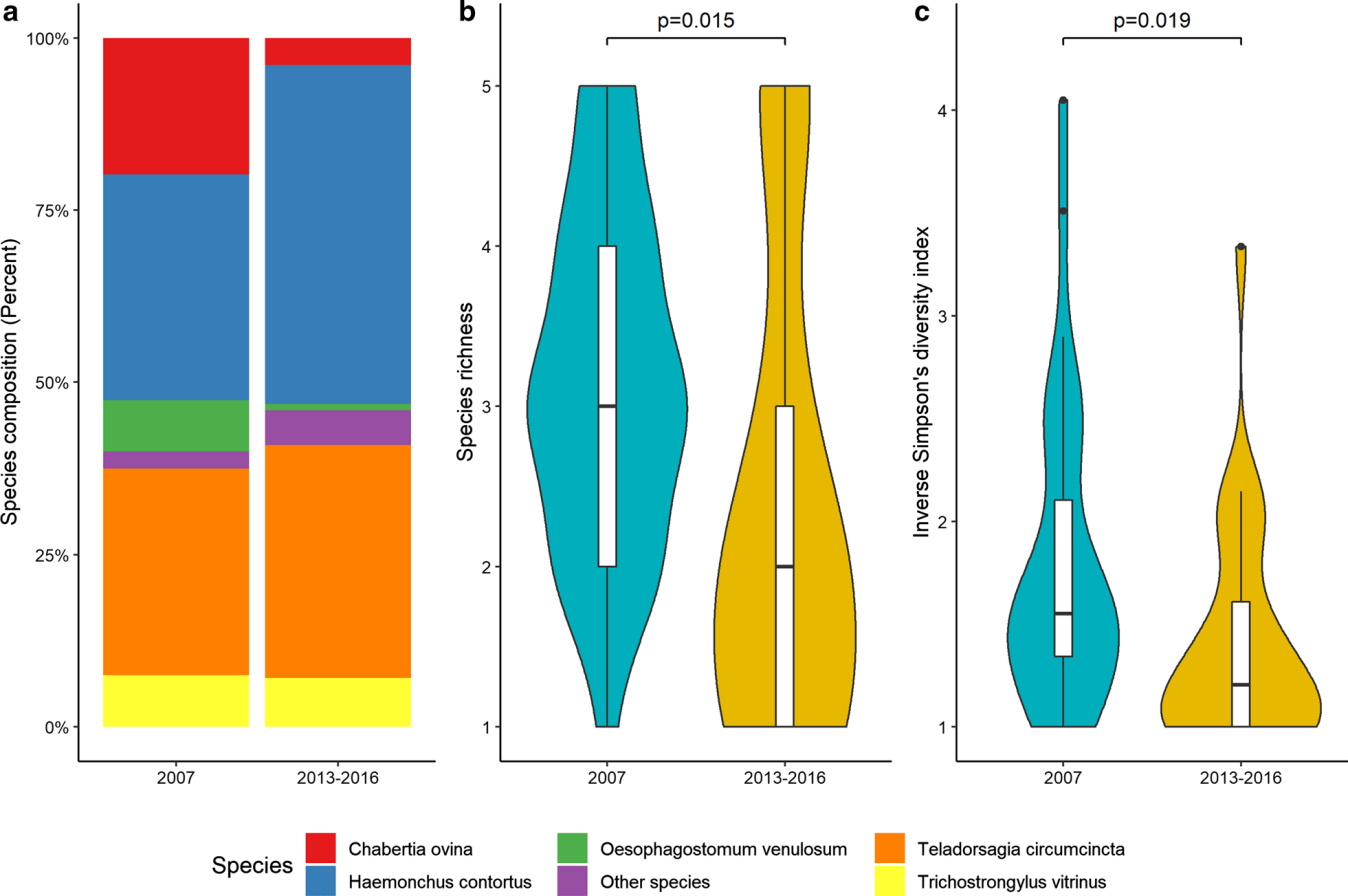
**a** Nemabiome composition for lambs for two periods (2007 *n* = 35, 2013–2016 *n* = 49), **b** Species richness and **c** Inverse Simpson's diversity index for each period. Boxplots inside the violin plots display the median

**Fig. 4.**
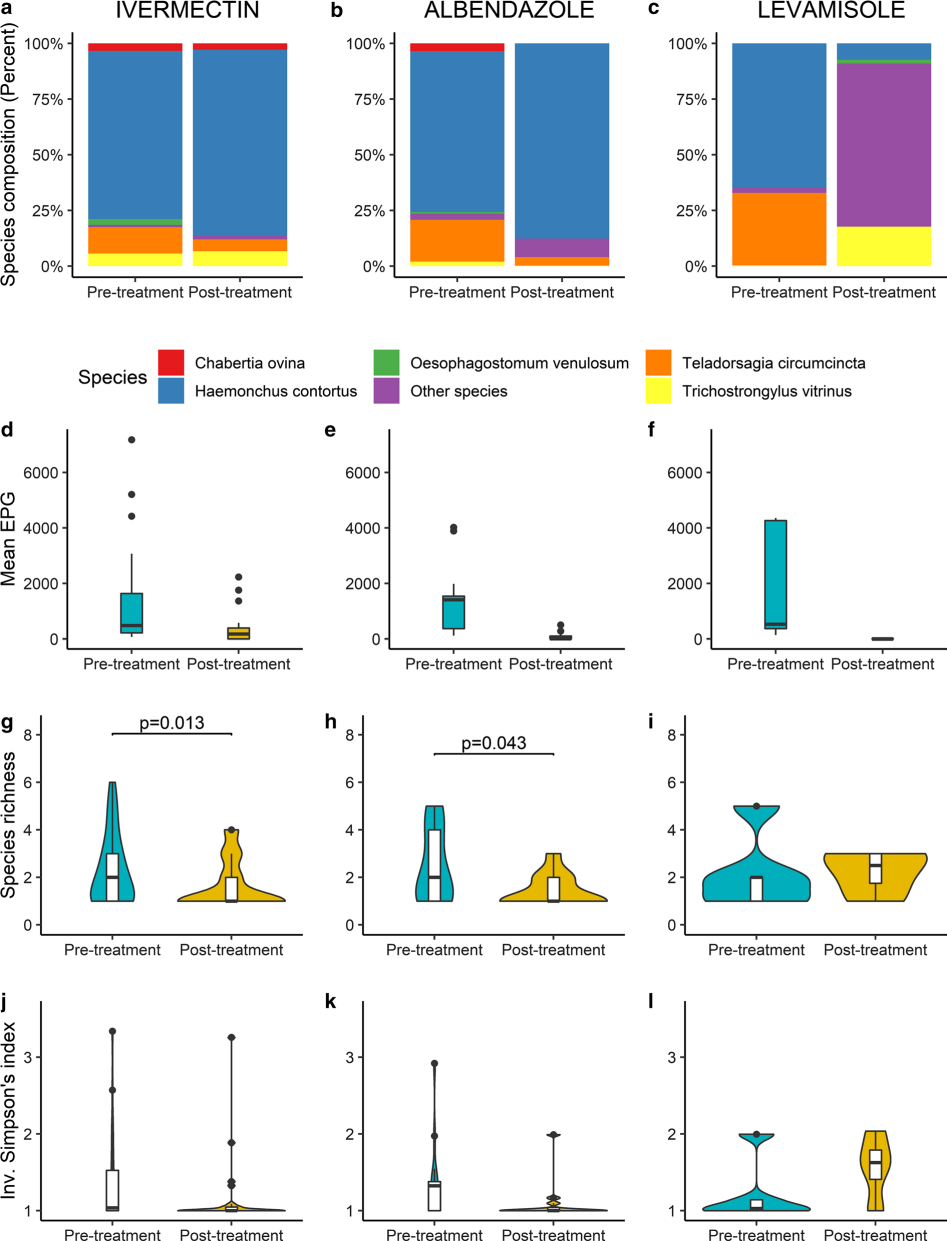
Nemabiome composition (**a**–**c**), EPG (**d**–**f**) and diversity indices: species richness (**g**–**i**). The shape of the violin plots in panel G indicate that anthelmintic treatment will have a larger effect for individuals with higher species richness prior to treatment. Inverse Simpson's index (**j**–**l**) prior to and after anthelmintic treatment for ivermectin (*n* = 25) in the left column, albendazole (*n* = 14) in the middle column and levamisole (*n* = 5) in the right column. Boxplots inside the violin plots display the median

All statistical analyses on community structure were performed in R v4.0.2 [31]. Species richness was calculated by summing up the number of helminth species per individual. To investigate helminth community diversity and whether it was dominated by a few species, the inverse Simpson’s diversity index was calculated using Vegan package [32]. The value of Simpson's D ranges from 0 to 1, with 0 representing infinite diversity and 1 representing no diversity, so the larger the value of D, the lower the diversity. For this reason, Simpson's index is expressed as its inverse (1/D). We used linear models (LM) for Figs. 2 and 3, and generalized mixed models (GLMM), with a repeated measure design, was used to assess the effects on the nemabiome composition after anthelmintic treatment (Fig. 4g–l), using respective diversity indices as the response variable, treatment as a fixed factor and farm as a random structure. GLMM was calculated using the MCMCglmm package [33]. Using a Bayesian framework is robust for analyzing data with unbalanced sample size groups. Run parameters are presented in Table 2. Post-treatment larval culture samples for two IVM farms were omitted because of successful treatment (Fig. 4j); hence, no nematode DNA was to be analyzed. Data were visualized with ggplot2 [34].

## Results

### Species diversity

The 159,224 high-quality reads were assembled into OTUs, with on average 1,008 reads per sample (range 9–1885 prior to filtering). After filtering, we identified 16 nematode species, based on OTU identification, from 158 sheep, of which 13 had a NCBI BLAST query cover > 99% (Table 1). Of the 158 larval culture samples, all except 2 collected post-treatment produced nematode OTUs. Of these, the five most common species consisted of roughly 97% of the reads for both adult sheep and lambs (Fig. 2; Table 1). The species in the stacked bar plots in Figs. 2a, 3a and 4a–c are calculated using fractions of the total number of reads per sample. Thus, each sample has equal representation in the figures. On average, adults were infected by fewer species compared to lambs (species richness, Fig. 2b) (LM, F_(1.110)_  = 2.721, *P* = 0.0036). However, the species composition showed that the nematode community was dominated by a fewer species among ewes compared to lambs (inverse Simpson’s diversity index, Fig. 2c) (LM, F_(1.110)_  = 9.165, *P* = 0.0063).

**Table 1:** Identification of species from OTUs using BLAST (basic local alignment search tool)

**Best species match from BLAST search**	**Query cover**	**Identity**	**Number of samples**	**OTU Sequence length**	**Number of sequence reads**
*Chabertia ovina*	100	100	37	283	8,418
*Haemonchus contortus*	100	100	127	281	102,110
*Teladorsagia circumcincta*	100	99	80	296	28,293
*Trichostrongylus vitrinus*	100	100	40	288	10,311
*Oesophagostomum venulosum*	99	100	20	308	3,288
*Bunostomum trigonocephalum*	100	100	3	281	199
*Cooperia curticei*	100	99	1	292	800
*Cooperia oncophora*	100	100	5	291	1,290
*Coronocyclus coronatus*	100	99	1	281	735
*Cylicocyclus nassatus*	100	98	10	370	1,294
*Cylicostephanus minutus*	99	100	3	266	200
*Cystocaulus ocreatus*	100	99	3	418	85
*Muellerius capillaris*	100	100	2	455	35
*Ostertagia leptospicularis*	100	99	1	289	66
*Ostertagia ostertagi*	100	100	3	288	364
*Varestrongylus eleguneniensis*	94	100	2	411	23

The top five species represent 97% of the reads. Nematodes were identified in 156 (99%) out of 158 larval culture samples. The most common species was *Haemonchus contortus*, which was identified in 127 (80%) of the positive samples and was also represented by most of the sequence reads across all samples. The ITS2 fragments for all OTUs varied in length from 266 to 455 bp

### Nemabiome composition

For lambs, we tested larval culture samples from two time periods, 2007 and 2013–2016, while samples for adults were only collected in 2013–2016. Among the lambs, *Chabertia ovina* decreased and *Haemonchus contortus* increased in frequency compared with adults. The species richness in lambs between the two time periods differed between the time periods (LM, F_(1.59)_ = 6.295, *P* = 0.0149). There was also a higher dominance among a few species in 2013–2016 than in 2007, based on the difference in inverse Simpson’s diversity index between the years according to a linear model (LM, F_(1.59)_ = 5.82, *P* = 0.019) (Fig. 3).

### Influence of anthelmintic treatment

Treatment with IVM was most effective against *Oesophagostomum venulosum*, ABZ was effective against *C. ovina* and *Trichostrongylus vitrinus*, while LEV showed high efficacy against *Teladorsagia circumcincta* and also had good effect against *H. contortus* (Fig. 4a–c). Furthermore, the post-treatment EPG was lower for all anthelmintic substances (Fig. 4d–f), and especially for LEV where no nematode eggs were found after treatment. In contrast, eggs remained in 17/26 (65%) and 7/13 (54%) of the groups treated with IVM and ABZ, respectively. Post-treatment lowered the species richness in IVM or ABZ treated sheep, (Fig. 4d, e) while no change in species dominance was detected (Fig. 4j–k). For LEV, the treatment was highly effective and the EPG in post-treated sheep was lower than 50 (the detection limit of the McMaster) for all farms (*n* = 5). Still, nemabiome data were generated, and the low number of parasites remaining notably changed in species composition although it did not notably affect species richness or species dominance (Fig. 4i–l). See Table 2 for detailed summary statistics.

**Table 2:** Output from MCMCglmm testing of the impact of each of the three anthelmintic drug treatments (IVM = ivermectin, ABZ = albendazole, LEV = levamisole) on species richness and inverse Simpson’s diversity index (see also Fig. 4g–l) for farms sampled before and after anthelmintic treatment

**Pre- vs. post-treatment**	**Anthelmintics**	**Posterior mean**	**Lower 95% CI**	**Upper 95% CI**	**Effective sample size**	**pMCMC**^**1**^	
Species richness	IVM	− 0.6567	− 1.1476	− 0.1682	9914	0.0128*	Fig. 4g
Species richness	ABZ	− 0.98885	− 2.00249	− 0.08799	9488	0.043*	Fig. 4h
Species richness	LEV	0.2294	− 1.7207	2.1984	10000	0.7696	Fig. 4i
Inv Simpson's index	IVM	− 0.20087	− 0.48078	0.07807	10000	0.165	Fig. 4j
Inv Simpson's index	ABZ	− 0.25936	− 0.63469	0.08315	10000	0.145	Fig. 4k
Inv Simpson's index	LEV	0.3575	− 0.3554	1.1646	9623	0.289	Fig. 4l

^1^Statistically significant fixed effects are marked with * (*P* < 0.05)

Prior settings: R (V = 2, nu = 0.2); G (V = 2, nu = 0.02)

Run settings: burn-in  =  50,000; iterations  =  100,000; thinning interval  =  10

As an example, untreated sheep were infected with on average 0.66 fewer species compared to IVM treated sheep. No such effect was found in species richness for LEV, nor for any of the inverse Simpsons’ indices

## Discussion

This study, for the first time to our knowledge, investigated the nemabiome in 158 larval culture samples from 61 commercial sheep farms in Sweden. The aims were to find out how nematode community structure (species composition and diversity) is influenced: (i) by host age (in ewes and lambs), (ii) in the short term following recent anthelmintic treatment with ABZ, IVM or LEV and (iii) longitudinally in samples collected several years apart. For this we used DNA extracted from composite larval cultures which then were analyzed using a PacBio NGS pipeline generating sequence data (amplicons) that were clustered into OTUs with a specifically designed analysis framework developed for eukaryotes (SCATA) [35–37]. We identified five common OTUs representing 97% of the reads, among which *H. contortus* and *T. circumcincta* were the two dominant species. There were no significant differences in nemabiomes post-treatment between ewes and lambs. It can be argued that a limitation is that the number of samples differed. The lambs across both time periods and the adults were *n* = 84 and *n* = 74, respectively. In contrast, use of anthelmintics had a short-term dramatic effect, but not on the nemabiome in post-treatment larval culture samples collected several years apart, indicating there was no long-term effect.

In total, 16 OTUs were identified, five of which are well-known and globally distributed strongyle nematodes associated with sheep, i.e. *C. ovina, H. contortus, T. circumcincta, Trichostrongylus vitrinus* and *Oesophagostomum venulosum*. This group of the big five constituted the majority of reads (97%). All of these had a match of ≥ 99 to 100 in query cover and identity according to BLAST searches and were therefore considered valid species. We also recognized five other species reported from sheep with the same confidence in most cases (Table 1). These were represented by two strongylids, *Bunostomum trigonocephalum* and *Cooperia curticei*, plus three metastrongylids (small lungworms): *Cystocaulus ocreatus, Muellerius capillaris* and a species related to *Varestrongylus eleguneniensis.* All had an identity of ≥ 99 and with the exception of the match to *Varestrongylus eleguneniensis* and a query cover of 100%. However, combining these five represented < 1% of the total reads, and they were only found in a few samples. This indicates that the epidemiological consequences and clinical impact of these parasites are probably of less importance for sheep raised under Swedish conditions. With the exception of *T. colubriformis*, which was absent in our data set, we recognized all eight major species found in UK sheep based on data generated with a similar approach [19]. However, the relative abundance of the distinctive species differed. In our study five species dominated the nemabiomes with *H. contortus* being more prevalent in Swedish compared to UK sheep. It is possible that the observed differences in the nemabiomes are related to differences in climatic conditions. Thus, microclimatic influences would be worthwhile to investigate especially in light of the possible impact of climate change.

In addition to the above species, we identified five species usually not found in sheep. Among these, three species are mainly linked to cattle: *Cooperia oncophora, Ostertagia leptospicularis* and *O. ostertagi* [2]. We also identified four cyathostomins associated with equids showing high query cover and identity (98% to 100%), indicating that these findings were valid. To the best of our knowledge, transmission of equid nematodes to sheep does not occur. Thus, it cannot be excluded that these findings were due to a trace contamination at either the DNA or parasitological level, especially as the number of samples was low (Table 1).

In contrast, opportunities for cross-infections between cattle and sheep nematodes have been studied through experimental cross-infections [38]. It was shown that both *Cooperia spp.* and *Ostertagia* spp*.* can infect lambs; however, there are varying degrees of host specificity in these genera, with *C. oncophora* and *O. ostertagi* being more adapted to cattle than to sheep. However, as stated by Herlich [39], it cannot be precluded that small numbers of *O. ostertagi* may attain sexual maturity in sheep under natural grazing conditions, which is in line with our findings.

Interestingly, we recognized no species primarily associated with wildlife., However, there are generalists known to infect a wide range of wildlife ungulates in Europe [7]. Not least, roe deer in Spain [40], Italy [41], Turkey [42] and France [43] are known to be infected with species identified herein. Among these, particularly *H. contortus* is the most pathogenic nematode of sheep in Sweden [3] and is therefore of major interest. Although, roe deer are abundant in Sweden, knowledge about their nemabiome and its role as a reservoir of livestock parasites is presently unknown. Thus, this needs to be studied in the future, especially with a focus on *H. contortus,*, especially in the face of climatic change, and its propensity to develop resistance to anthelmintic drugs and the possibility of roe deer acting as a reservoir and spreading it widely. Since it is known that there are cryptic species that appear identical but are genetically distinct, particularly among members of the superfamily Trichostrongyloidea that hybridize [44], we are convinced that nemabiome analysis is well suited for this.

Clearly, the culturing conditions for nematode eggs can also have an impact on the nemabiome composition [45]. For example, it has been shown that fewer eggs of *T. circumcincta* develop to the third stage than for *T. colubriformis*. Also, the development of larvae of other strongyles is heavily influenced by refrigeration [45]. Extracting DNA from fecal matter or from larval cultures both had advantages and disadvantages. (i) DNA from fecal samples would contain DNA from all eggs and might contain inhibitors while fecal samples containing few parasite eggs might lead to an underestimation of species diversity and contain inhibitors. (ii) Larval culture samples will contain more parasite larvae, while the hatchability might be impared because of storage. Besides from the input material, the number of recognized OTUs is also influenced by (i) primer design and PCR conditions, (ii) the choice of NGS platform and bioinformatics pipeline for OTU clustering and recognition and, not least, (iii) available sequence information in the sequence databases. In this study we took advantage of the commonly used primers NC1 and NC2 and compared the obtained sequence with information in NCBI. In line with previous studies, these universal primers spanning the second (ITS2) internal transcribed spacer in the nuclear ribosomal DNA array not only amplify a wide range of livestock genera of nematodes of veterinary interest (i.e. *Bunostomum, Chabertia, Cooperia, Dictyocaulus, Haemonchus, Nematodirus, Oesophagostomum, Ostertagia, Varestrongylus, Teladorsagia,* and *Trichostrongylus*), but also provide suitable genetic markers for species delimitation (for a review see Gasser et al. 2008). In our study, most of these genera were found as well as five additional genera (Table 1). Still, it is unclear where to define the cut-off for discriminating between nematode species, as genetic isolation is generally used to define species boundaries rather than DNA differences [46]. Nevertheless, in agreement with previous studies on the nemabiome in livestock and horses [16–20], a cut-off identity threshold ≥ 99 seems reasonable. However, as shown in our study, the query cover also needs to be considered. Still, when targeting the ITS region it has been shown that PacBio sequencing better reflects the composition of fungal communities than Illumina MiSeq because of sequence length variation [37]. However, in this study the ITS2 for the different OTUs varied between 266 and 455 bp (Table 1). This might explain why metastrongyles (411–455 bp) were detected in a few samples. To what extent the relative species abundance of nemabiome communities is affected by sequence length variation needs to be explored in more detail. Further investigations also need to address factors that may introduce sequencing bias.

We observed an effect in relation to host age, and long-term effects were observed when we compared the larval culture samples collected in 2007 and 2013–2016 (Figs. 2 and 3). The observation that adults had less diversity than lambs is expected as adults can build up resistance to some parasites. It is also illustrated by that some species dominated the nemabiomes in adults. It is clear that use of anthelmintics drastically influenced the nemabiome composition (Fig. 4). In this context, it is important to consider that the use of the tested drugs has changed during the last decades. In Sweden, the drug of first choice whenever *H. contortus* is present has, from the 1960s until recently, changed from ABZ to IVM [47]. However, with emerging evidence for double-resistant *H. contortus* strains, this practice has changed. Today, LEV is increasingly used, but currently only on farms that respond adequately to neither IVM nor ABZ. Interestingly, our data show that the identified nematode species responded differently to these drugs. Of particular practical interest is that both IVM and ABZ were unable to control *H. contortus* on several farms, whereas LEV still had a reasonable efficacy. However, although there were always zero egg counts post-treatment with LEV, it is evident that in particular *H. contortus* survived at a low level. Likewise, *T. circumcincta,* which is the second most important nematode in Swedish sheep, survived treatment with either ivermectin or ABZ, but then to a lesser extent than *H. contortus*, whereas not at all after LEV treatment. This is partly in contrast to the situation reported several years ago in a global context, when resistance to LEV was widely prevalent among several trichostrongylid nematodes of sheep [48]. Overall, we believe the observed patterns reflect the current anthelmintic resistance situation in GIN of sheep in Sweden. Of major practical concern is whether LEV-resistant *H. contortus* will appear with increasing use of this drug. In countries with more intense sheep production (e.g. New Zealand), the use of LEV has decreased in favor of other drugs or drug combinations with increased reports of resistance [49]. In the past when LEV was used as a single compound in sheep, high levels of resistance emerged, like for most other commonly used anthelmintics [50]. In light of this, our observations showing low levels of surviving *H. contortus* are of great concern.

## Conclusions

In our study, the nemabiome approach proved to be a practical method of studying nematode community diversity in sheep and how it is influenced by factors such as host age and the most recent use of anthelmintic treatment. Clearly, by using another molecular approach (droplet digital PCR), estimates of sheep nematode diversity were underestimated [14]. From this study it is clear that the effects of recent anthelmintic treatment of gastrointestinal parasites in sheep can be investigated at a detailed level with high-throughput sequencing.

## Supplementary Information

**Additional file 1: Table S1.** Species identification without 1% cut-off. **Table S2.** MCMCglmm analysis without 1% cut-off. **Figure S1**. Scatterplot with number of sequences per sample. **Figure S2.** Nemabiome composition for adults and lambs without 1% cut-off. **Figure S3.** Nemabiome composition for lambs for two periods using data without 1% cut-off. **Figure S4.** Nemabiome composition and diversity indices without 1% cut-off.

## Data Availability

The datasets used and/or analyzed during the current study are available from the corresponding author on reasonable request. The raw ITS2 data are available in the BioStudies database (http://www.ebi.ac.uk/biostudies) under accession number S-BSST527.

## References

[1] Sutherland I, Scott I (2010). Gastrointestinal nematodes of sheep and cattle : biology and control.

[2] Andersson RC (1992). Nematode parasites of vertebrates - their development and transmission.

[3] Höglund J, Elmahalawy ST, Halvarsson P, Gustafsson K. Detection of Haemonchus contortus on sheep farms increases using an enhanced sampling protocol combined with PCR based diagnostics. Vet Parasitol X. Elsevier B.V.; 2019;2:100018. 10.1016/j.vpoa.2019.100018 34311930

[4] Morgan ER, van Dijk J (2012). Climate and the epidemiology of gastrointestinal nematode infections of sheep in Europe. Vet Parasitol. Elsevier.

[5] Wolstenholme AJ, Fairweather I, Prichard R, Von Samson-Himmelstjerna G, Sangster NC (2004). Drug resistance in veterinary helminths. Trends Parasitol..

[6] Mcrae KM, Stear MJ, Good B, Keane OM (2015). The host immune response to gastrointestinal nematode infection in sheep. Parasite Immunol..

[7] Walker JG, Morgan ER (2014). Generalists at the interface: Nematode transmission between wild and domestic ungulates. Int J Parasitol Parasites Wildl..

[8] Taylor MA (2010). Parasitological examinations in sheep health management. Small Rumin Res..

[9] Roeber F, Hassan EB, Skuce P, Morrison A, Claerebout E, Casaert S (2017). An automated, multiplex-tandem PCR platform for the diagnosis of gastrointestinal nematode infections in cattle: an Australian-European validation study. Vet Parasitol..

[10] Charlier J, Morgan ERR, Rinaldi L, Van Dijk J, Demeler J, Höglund J (2014). Practices to optimise gastrointestinal nematode control on sheep, goat and cattle farms in Europe using targeted (selective) treatments. Vet Rec.

[11] Rose H, Rinaldi L, Bosco A, Mavrot F, De Waal T, Skuce P, et al. Widespread anthelmintic resistance in European farmed ruminants: a systematic review. Vet. Rec. British Veterinary Association; 2015. p. 546. 10.1136/vr.102982 25762583

[12] Gasser RB, Bott NJ, Chilton NB, Hunt P, Beveridge I (2008). Toward practical, DNA-based diagnostic methods for parasitic nematodes of livestock - Bionomic and biotechnological implications. Biotechnol Adv..

[13] Hunt PW, Lello J (2012). How to make DNA count: DNA-based diagnostic tools in veterinary parasitology. Vet Parasitol..

[14] Elmahalawy ST, Halvarsson P, Skarin M, Höglund J (2018). Droplet digital polymerase chain reaction (ddPCR) as a novel method for absolute quantification of major gastrointestinal nematodes in sheep. Vet Parasitol..

[15] Roeber F, Jex AR, Campbell AJD, Campbell BE, Anderson GA, Gasser RB (2011). Evaluation and application of a molecular method to assess the composition of strongylid nematode populations in sheep with naturally acquired infections. Infect Genet Evol..

[16] Avramenko RW, Redman EM, Lewis R, Yazwinski TA, Wasmuth JD, Gilleard JS (2015). Exploring the Gastrointestinal “Nemabiome”: Deep Amplicon Sequencing to Quantify the Species Composition of Parasitic Nematode Communities. PLoS ONE..

[17] Avramenko RW, Bras A, Redman EM, Woodbury MR, Wagner B, Shury T (2018). High species diversity of trichostrongyle parasite communities within and between Western Canadian commercial and conservation bison herds revealed by nemabiome metabarcoding. Parasites Vectors..

[18] Scott H, Gilleard J, Jelinski M, Barkema H, Redman E, Avramenko R (2019). Prevalence, fecal egg counts, and species identification of gastrointestinal nematodes in replacement dairy heifers in Canada. J Dairy Sci..

[19] Redman E, Queiroz C, Bartley DJ, Levy M, Avramenko RW, Gilleard JS (2019). Validation of ITS-2 rDNA nemabiome sequencing for ovine gastrointestinal nematodes and its application to a large scale survey of UK sheep farms. Vet Parasitol..

[20] Mitchell CJ, O’Sullivan CM, Pinloche E, Wilkinson T, Morphew RM, McEwan NR (2019). Using next-generation sequencing to determine diversity of horse intestinal worms: Identifying the equine ŉemabiome’. J Equine Sci..

[21] Fichot EB, Norman RS (2013). Microbial phylogenetic profiling with the Pacific Biosciences sequencing platform. Microbiome..

[22] McKenna PB (1997). Anthelmintic treatment and the suppression of egg production in gastro-intestinal nematodes of sheep and cattle: Fact or fallacy?. N Z Vet J..

[23] Ljungström S, Melville L, Skuce PJPJ, Höglund J (2018). Comparison of four diagnostic methods for detection and relative quantification of Haemonchus contortus eggs in feces samples. Front Vet Sci. Frontiers.

[24] McKenna PB (1997). Anthelmintic treatment and the suppression of egg production in gastro-intestinal nematodes of sheep and cattle: Fact or fallacy?. N Z Vet J..

[25] Gasser RB, Chilton NB, Hoste H, Beveridge I. Rapid sequencing of rDNA from single worms and eggs of parasitic helminths. Nucleic Acids Res [Internet]. 1993;21:2525–6. Available from: https://academic.oup.com/nar/article-lookup/doi/10.1093/nar/21.10.2525 10.1093/nar/21.10.2525 PMC309567 8506152

[26] Ihrmark K, Bödeker ITM, Cruz-Martinez K, Friberg H, Kubartova A, Schenck J, et al. New primers to amplify the fungal ITS2 region - evaluation by 454-sequencing of artificial and natural communities. FEMS Microbiol Ecol [Internet]. 2012;82:666–77. Available from: https://academic.oup.com/femsec/article-lookup/doi/10.1111/j.1574-6941.2012.01437.x 10.1111/j.1574-6941.2012.01437.x 22738186

[27] Lindahl BD, Nilsson RH, Tedersoo L, Abarenkov K, Carlsen T, Kjøller R, et al. Fungal community analysis by high-throughput sequencing of amplified markers - a user’s guide. New Phytol [Internet]. 2013;199:288–99. Available from: http://doi.wiley.com/10.1111/nph.12243 10.1111/nph.12243 PMC3712477 23534863

[28] Laehnemann D, Borkhardt A, McHardy AC (2016). Denoising DNA deep sequencing data—high-throughput sequencing errors and their correction. Brief Bioinform..

[29] Edgar RC. Search and clustering orders of magnitude faster than BLAST. Bioinformatics [Internet]. 2010;26:2460–1. Available from: https://academic.oup.com/bioinformatics/article-lookup/doi/10.1093/bioinformatics/btq461 10.1093/bioinformatics/btq461 20709691

[30] Kõljalg U, Nilsson RH, Abarenkov K, Tedersoo L, Taylor AFS, Bahram M, et al. Towards a unified paradigm for sequence-based identification of fungi. Mol Ecol [Internet]. 2013;22:5271–7. Available from: http://doi.wiley.com/10.1111/mec.12481 10.1111/mec.12481 24112409

[31] R Core Team. R: A Language and Environment for Statistical Computing [Internet]. Vienna, Austria; 2020. Available from: https://www.r-project.org

[32] Oksanen J, Blanchet FG, Friendly M, Kindt R, Legendre P, McGlinn D, et al. vegan: Community Ecology Package [Internet]. 2019. Available from: https://cran.r-project.org/package=vegan

[33] Hadfield JD. MCMC Methods for Multi-Response Generalized Linear Mixed Models: The {MCMCglmm} {R} Package. J Stat Softw [Internet]. 2010;33:1–22. Available from: http://www.jstatsoft.org/v33/i02/

[34] Wickham H. ggplot2: Elegant Graphics for Data Analysis [Internet]. Springer-Verlag New York; 2016. Available from: https://ggplot2.tidyverse.org

[35] Kyaschenko J, Clemmensen KE, Hagenbo A, Karltun E, Lindahl BD (2017). Shift in fungal communities and associated enzyme activities along an age gradient of managed Pinus sylvestris stands. ISME J..

[36] Sterkenburg E, Clemmensen KE, Lindahl BD, Dahlberg A (2019). The significance of retention trees for survival of ectomycorrhizal fungi in clear-cut Scots pine forests. J Appl Ecol..

[37] Castaño C, Berlin A, Brandström Durling M, Ihrmark K, Lindahl BD, Stenlid J, et al. Optimized metabarcoding with Pacific Biosciences enables semi‐quantitative analysis of fungal communities. New Phytol [Internet]. Wiley; 2020 [cited 2020 Jul 8];nph.16731. Available from: https://onlinelibrary.wiley.com/doi/abs/10.1111/nph.16731 10.1111/nph.16731 32531109

[38] Borgsteede FHM (1981). Experimental Cross-Infections with Gastrointestinal Nematodes of Sheep and Cattle. Zeitschrift für Parasitenkd..

[39] Herlich H (1971). Infection Dynamics of the Cattle Parasite, Ostertagia ostertagi, in Sheep. Proc Helminthol Soc Wash..

[40] Pato FJ, Vázquez L, Díez-Baños N, López C, Sánchez-Andrade R, Fernández G (2013). Gastrointestinal nematode infections in roe deer (Capreolus capreolus) from the NW of the Iberian Peninsula: Assessment of some risk factors. Vet Parasitol..

[41] Zaffaroni E, Manfredi MT, Citterio C, Sala M, Piccolo G, Lanfranchi P (2000). Host specificity of abomasal nematodes in free ranging alpine ruminants. Vet Parasitol..

[42] Bolukbas CS, Gurler AT, Beyhan YE, Acici M, Umur S (2012). Helminths of roe deer (Capreolus capreolus) in the Middle Black Sea Region of Turkey. Parasitol Int. Elsevier.

[43] Ferté H, Cléva D, Depaquit J, Gobert S, Léger N (2000). Status and origin of Haemonchinae (Nematoda: Trichostrongylidae) in deer: A survey conducted in France from 1985 to 1998. Parasitol Res..

[44] Wyrobisz A, Kowal J, Nosal P (2016). Insight into species diversity of the Trichostrongylidae Leiper, 1912 (Nematoda: Strongylida) in ruminants. J Helminthol..

[45] Donald AD, Waller PJ, Dobson RJ, Axelsen A (1980). The effect of selection with levamisole on benzimidazole resistance in *Ostertagia* spp. of sheep. Int J Parasitol..

[46] Nadler S. Species delimitation and nematode biodiversity: phylogenies rule. Nematology. 2002;4:615–25. https://brill.com/view/journals/nemy/4/5/article-p615_10.xml

[47] Höglund J, Gustafsson K, Ljungström BL, Skarin M, Varady M, Engström F (2015). Failure of ivermectin treatment in Haemonchus contortus infected-Swedish sheep flocks. Vet Parasitol Reg Stud Reports..

[48] Sangster NC, Bjorn H (1995). Levamisole resistance in Haemonchus contortus selected at different stages of infection. Int J Parasitol. Pergamon.

[49] McKenna PB (1990). The use of benzimidazole-levamisole mixtures for the control and prevention of anthelmintic resistance in sheep nematodes: an assessment of their likely effects. N Z Vet J..

[50] Waghorn TS, Leathwick DM, Rhodes AP, Lawrence KE, Jackson R, West DM (2006). Prevalence of anthelmintic resistance on sheep farms in New Zealand. N Z Vet J..

