# Supplementary material for: Sheep nemabiome diversity and its response to anthelmintic treatment in Swedish sheep herds
Source: Parasit Vectors. 2021 Feb 18;14:114. doi: 10.1186/s13071-021-04602-y (PMC7890823; doi:10.1186/s13071-021-04602-y)
Supplement: Supplementary file 1 — Additional file 1: Table S1. Species identification without 1% cut-off. Table S2. MCMCglmm analysis without 1% cut-off. Figure S1. Scatterplot with number of sequences per sample. Figure S2. Nemabiome composition for adults and lambs without 1% cut-off. Figure S3. Nemabiome composition for lambs for two periods using data without 1% cut-off. Figure S4. Nemabiome composition and diversity indices without 1% cut-off. [file 13071_2021_4602_MOESM1_ESM.docx]

**Additional file**

**Table S1.** Identification of species from OTUs using BLAST without a 1% sequence threshold for accepting a species. The top five species represent 97% of the reads. Nematodes were identified in 156 (99%) out of 158 samples. The most common species was *Haemonchus contortus*, which was identified in 135 (85%) of the positive samples. The ITS2 fragments varied in length from 266 to 512 bp.

| Best species match from BLAST search | Query cover | Identity | Number of samples | ITS2 OTU Sequence length (bp) |
| --- | --- | --- | --- | --- |
| *Chabertia ovina* | 100 | 100 | 48 | 283 |
| *Haemonchus contortus* | 100 | 100 | 135 | 281 |
| *Teladorsagia circumcincta* | 100 | 99 | 97 | 296 |
| *Trichostrongylus vitrinus* | 100 | 100 | 57 | 288 |
| *Oesophagostomum venulosum* | 99 | 100 | 32 | 308 |
| *Bunostomum trigonocephalum* | 100 | 100 | 3 | 281 |
| *Cooperia curticei* | 100 | 99 | 1 | 292 |
| *Cooperia oncophora* | 100 | 100 | 10 | 291 |
| *Coronocyclus coronatus* | 100 | 99 | 2 | 281 |
| *Coronocyclus labratus* | 100 | 100 | 32 | 367 |
| *Craterostomum acuticaudatum* | 86 | 100 | 1 | 370 |
| *Cylicocyclus nassatus* | 100 | 99 | 24 | 370 |
| *Cylicocyclus ultrajectinus* | 87 | 100 | 1 | 402 |
| *Cylicostephanus minutus* | 99 | 100 | 5 | 266 |
| *Cystocaulus ocreatus* | 100 | 99 | 5 | 418 |
| *Dictyocaulus viviparus* | 97 | 97 | 1 | 512 |
| *Muellerius capillaris* | 100 | 100 | 6 | 455 |
| *Nematodirus spathiger* | 100 | 99 | 9 | 280 |
| *Ostertagia leptospicularis* | 100 | 99 | 5 | 289 |
| *Ostertagia ostertagi* | 100 | 100 | 5 | 288 |
| *Protostrongylus hobmaieri* | 95 | 97 | 3 | 400 |
| *Varestrongylus eleguneniensis* | 94 | 100 | 3 | 411 |
| *Strongyloides fuelleborni fuelleborni* | 100 | 90 | 2 | 360 |
| *Trichostrongylus axei* | 100 | 93 | 1 | 290 |
| *Trichostrongylus retortaeformis* | 100 | 98 | 2 | 288 |
| *Triodontophorus serratus* | 86 | 100 | 1 | 384 |

**Table S2.** Output from MCMCglmm testing of the impact of each of the three anthelmintic drug treatments (IVM=ivermectin, ABZ=albendazole, LEV=levamisole) on species richness and inverse Simpson’s diversity index (see also Fig 4G-4L) for farms sampled before and after anthelmintic treatment without 1% threshold.

| **Post- and pre-treatment** | **Posterior mean** | **Lower 95% CI** | **Upper 95%Ci** | **Effective sample size** | **pMCMC^1^** |  |
| --- | --- | --- | --- | --- | --- | --- |
| Species richness IVM | 0.92261 | 0.01674 | 1.76027 | 10819 | 0.0366* | Fig S4G |
| Species richness ABZ | 2.1461 | 1.0375 | 3.2327 | 10691 | 0.0008*** | Fig S4H |
| Species richness LEV | 0.994394 | -1.699608 | 3.847177 | 9560 | 0.4242 | Fig S4I |
| Inverse Simpson IVM | 0.20262 | -0.06626 | 0.47498 | 10000 | 0.1320 | Fig S4J |
| Inverse Simpson BZ | 0.2544 | -0.08311 | 0.60872 | 10000 | 0.1430 | Fig S4K |
| Inverse Simpson LEV | -0.2207 | -0.9139 | 0.4719 | 10000 | 0.4858 | Fig S4L |
| ^1^Statistically significant fixed effects are marked with * and *** | | | | | | |
| Prior settings: | R (V=2, nu =0.2); G (V=2, nu = 0.02) | | | | | |
| Run settings: | Burn-in = 50,000; Iterations = 100,000; Thinning interval = 10 | | | | | |


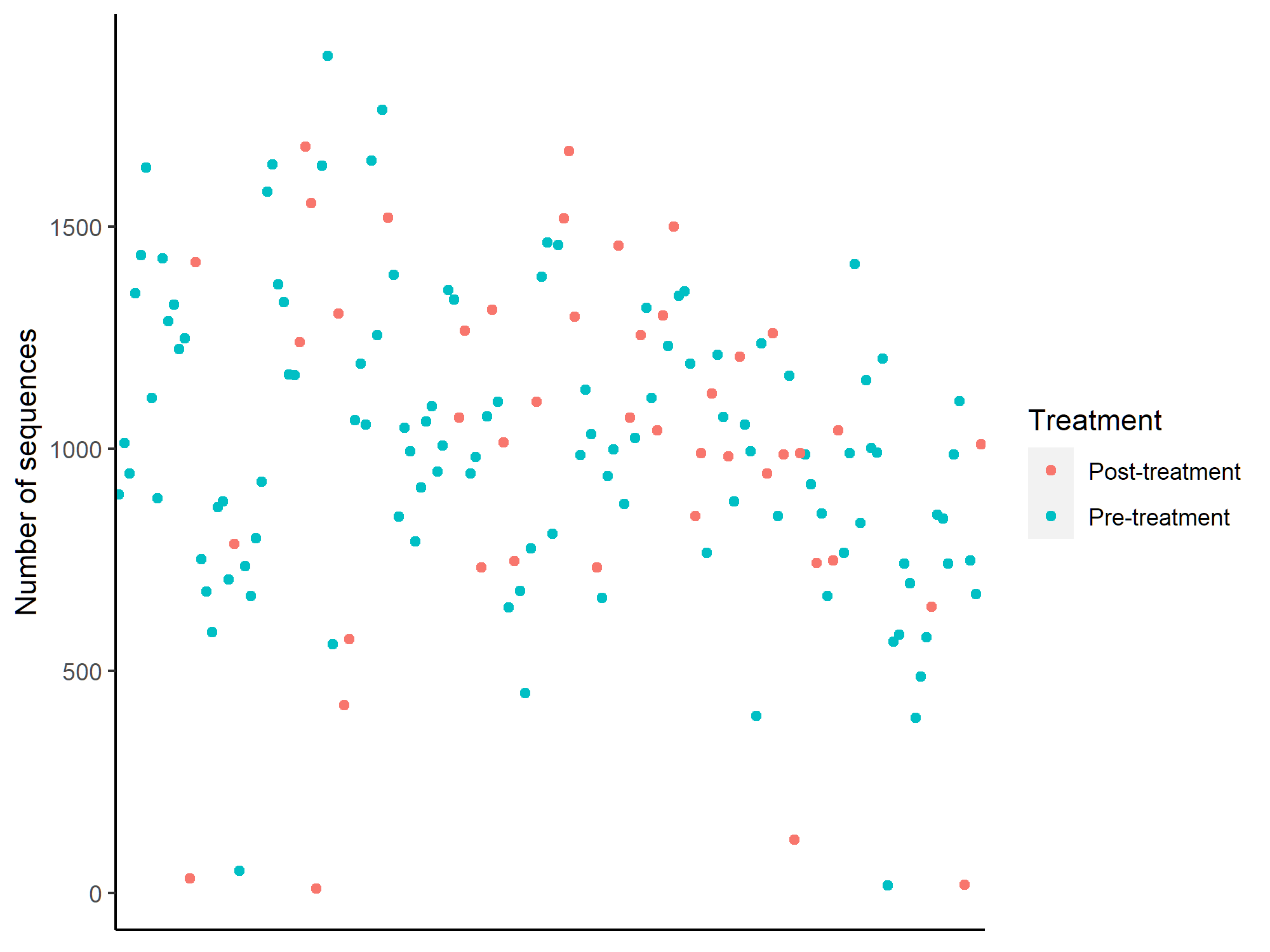


**Figure S1.** Number of sequences from the PacBio sequencing per sample divided on pre- and post treatment.


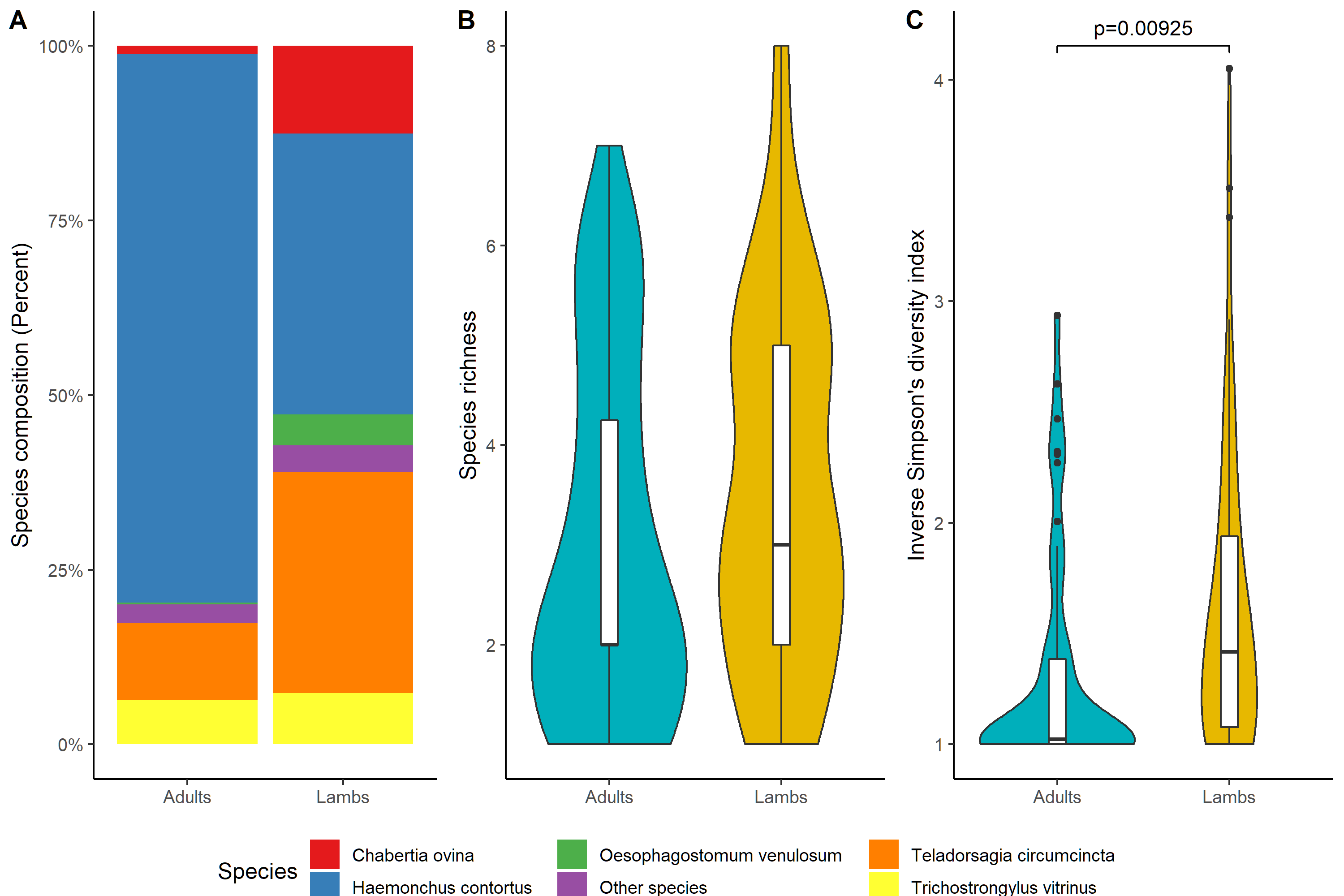
**Figure S2.** A) Nemabiome composition for adults and lambs without using 1% threshold for accepting a species. No difference in nematode species richness (B), but adults were dominated by a few species compared to lambs (C).


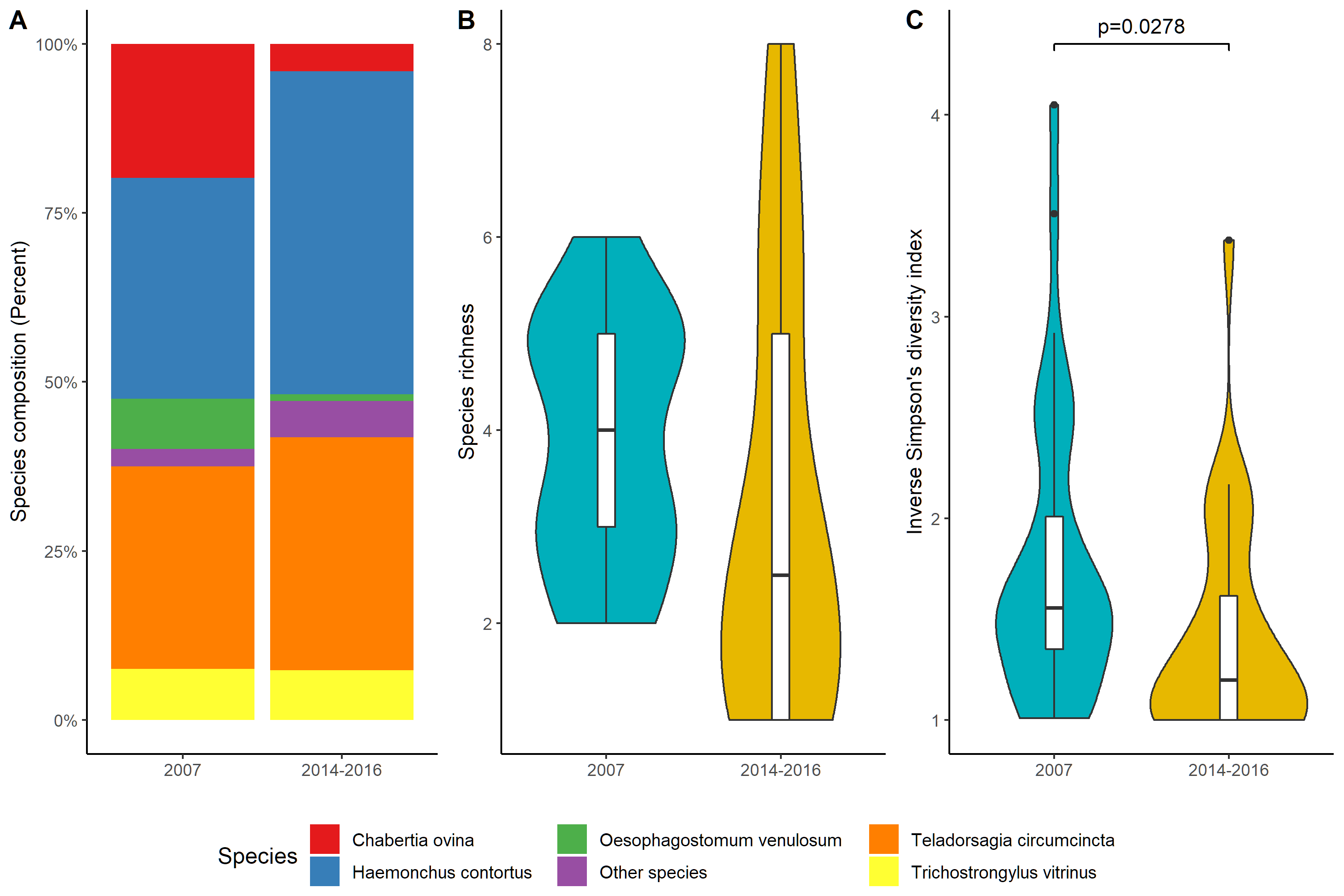


**Figure S3.** A) Nemabiome composition for lambs for two periods using data without 1% threshold for accepting a species. B) Species richness and C) Inverse Simpson´s diversity index for each period.


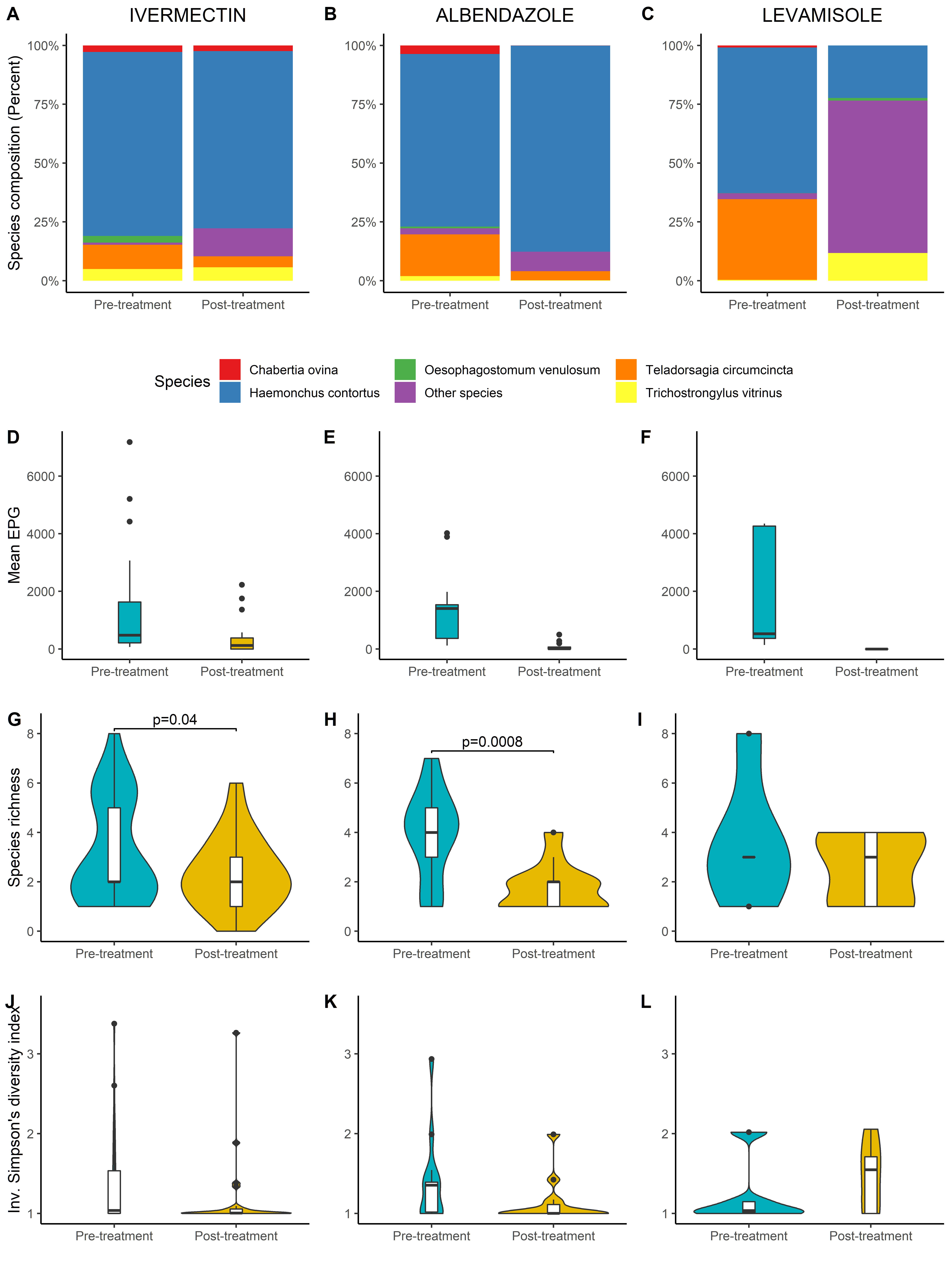


**Figure S4.** Nemabiome composition (A-C), EPG (D-F) and diversity indices; species richness (G-I) without a 1% threshold. The shape of the violin plots in panel G indicate that anthelmintic treatment will have a larger effect for individuals with higher species richness prior to treatment. Inverse Simpson´s index (J-L) prior and after anthelmintic treatment, for ivermectin (n=25) in left column, albendazole (n=14) in middle column and levamisole (n=5) in right column.
